# Dietary Pattern and Plasma BCAA-Variations in Healthy Men and Women—Results from the KarMeN Study

**DOI:** 10.3390/nu10050623

**Published:** 2018-05-15

**Authors:** Benedikt Merz, Lara Frommherz, Manuela J. Rist, Sabine E. Kulling, Achim Bub, Bernhard Watzl

**Affiliations:** 1 Department of Physiology and Biochemistry of Nutrition, Max Rubner-Institut, 76131 Karlsruhe, Germany; Manuela.Rist@mri.bund.de (M.J.R.); Achim.Bub@mri.bund.de (A.B.); Bernhard.Watzl@mri.bund.de (B.W.); 2 Department of Safety and Quality of Fruit and Vegetables, Max Rubner-Institut, 76131 Karlsruhe, Germany; Lara.Frommherz@mri.bund.de (L.F.); Sabine.Kulling@mri.bund.de (S.E.K.)

**Keywords:** KarMeN study, BCAA, dietary pattern, AAA, protein source

## Abstract

Branched-chain amino acids (BCAA) in plasma are discussed as risk factors for the onset of several diseases. Information about the contribution of the overall diet to plasma BCAA concentrations is controversial. Our objective was to investigate which dietary pattern is associated with plasma BCAA concentrations and whether other additional nutrients besides BCAA further characterize this dietary pattern. Based on the cross-sectional KarMeN study, fasting plasma amino acid (AA) concentrations, as well as current and habitual dietary intake were assessed in 298 healthy individuals. Using reduced rank regression, we derived a habitual dietary pattern that explained 32.5% of plasma BCAA variation. This pattern was high in meat, sausages, sauces, eggs, and ice cream but low in nuts, cereals, mushrooms, and pulses. The age, sex, and energy intake adjusted dietary pattern score was associated with an increase in animal-based protein together with a decrease in plant-based protein, dietary fiber, and an unfavorable fatty acid composition. Besides BCAA, alanine, lysine and the aromatic AA were positively associated with the dietary pattern score as well. All of these factors were reported to be associated with risk of type 2 diabetes and cardiovascular diseases before. Our data suggest that rather than the dietary intake of BCAA, the overall dietary pattern that contributes to high BCAA plasma concentrations may modulate chronic diseases risk.

## 1. Introduction

Diet is the only source of the essential branched-chain amino acids (BCAA) isoleucine, leucine, and valine in humans. Dietary sources of these proteinogenic amino acids (AA) are protein-containing foods, with meat and dairy being the main sources in omnivores [1,2]. Plasma BCAA concentrations are furthermore influenced via catabolism of muscle protein. Due to their higher muscle mass, men are reported to have higher BCAA levels compared to women. Further, obese compared to lean individuals, as well as individuals consuming a high-protein and/or high-energy diet show higher plasma BCAA concentrations [3].

There is insufficient and contradictory data on the contribution of diet to human plasma BCAA concentrations. Rietman et al. recently concluded that about 80% of ingested dietary BCAA reach the blood circulation [4], whereas studies investigating the kinetics of BCAA report less than 40% absorption rate [5,6,7]. In contrast, observational studies reported only weak correlations between dietary and plasma BCAA concentrations [1,8,9]. Furthermore, it is unclear whether plasma BCAA concentrations may reflect short-term or long-term dietary intake [4].

Several observational studies suggest that high plasma concentrations of BCAA are associated with an increased risk of insulin resistance, type 2 diabetes (T2D) or cardiovascular diseases (CVD) [1,3,10,11]. In contrast, some intervention studies reported independent positive associations of BCAA with risk of future diabetes, but information on the composition of the overall diet as the source of these essential AA is scarce. Other studies suggested that the observed associations between BCAA and insulin resistance or T2D are only of correlative but not causal nature [12,13,14]. Nonetheless, many questions remain as to whether these BCAA are causally related with the onset of these diseases, and if causal, what the underlying mechanisms may be. It is as well currently unclear whether specific BCAA-providing foods or dietary patterns determine circulating BCAA concentrations, or whether host metabolism is the primary driver [15,16]. In particular, information on dietary patterns that contribute to BCAA levels is lacking, although especially this information is highly relevant, because BCAA are usually consumed within a dietary pattern and not as isolated nutrients. Zheng et al. [1] reported a markedly attenuated association between BCAA intake and the observed risk of future diabetes when adjusting for total protein intake—hinting for a role of the overall diet instead of an isolated BCAA effect. Furthermore, Fontana et al. [17] stated that the AA composition of the diet rather than the total protein intake affects metabolic health.

The aim of this study was therefore to identify a dietary pattern that determines plasma BCAA concentrations, and to analyze whether further nutrients, such as other AA, are associated with this dietary pattern. We hypothesized that nutrients beyond BCAA may contribute to the observed increased disease risk of Western diets.

## 2. Materials and Methods 

### 2.1. Study Design

The Karlsruhe Metabolomics and Nutrition (KarMeN) Study is a cross-sectional study conducted at the Max Rubner-Institut in Karlsruhe, Germany, between 2011 and 2013, aiming to determine the impact of a number of factors on the human metabolome in healthy men and women. Study design and examination procedures are described in detail elsewhere [18]. In brief, a total of 312 voluntary individuals aged 18 to 80 years were recruited. Exclusion criteria were smoking, acute or regular medication including hormonal contraceptives for women, illness requiring treatment, supplement use, and additionally for women pregnancy or breast-feeding. Each individual visited the study center three times for a detailed characterization [18]. The study was conducted after approval of the local ethics committee (State Medical Council of Baden-Württemberg) and according to the guidelines of the Declaration of Helsinki. All participants gave written informed consent prior to study participation.

Participants were examined by trained study personnel according to standard operating procedures, and anthropometric, clinical and functional parameters were assessed [18]. On the second study center visit, participants provided a peripheral venous blood sample after an overnight fast of at least 10 h using 9 mL EDTA plasma tubes (S-Monovette, Sarstedt, Nümbrecht, Germany). Blood was centrifuged at 1850× *g* at 4 °C and aliquoted into small portions. In addition, serum samples (S-Monovette Z-gel, Sarstedt, Nümbrecht, Germany) were collected for standard clinical biochemistry analyses.

### 2.2. BCAA Measurement

A total of 21 AA (19 proteinogenic) were determined by an API 5500 Q-Trap mass spectrometer (AB Sciex Germany GmbH, Darmstadt, Germany) coupled to a Shimadzu Nexera UHPLC-system in fasting plasma samples using the Absolute IDQ™ p180 kit (Biocrates AG, Innsbruck, Austria). A 20 µL plasma aliquot was used for each extraction. Protein precipitation and subsequent derivatization with phenyl isothiocyanate was performed according to the manufacturer’s protocol prior to MS-detection. For chromatographic separation of AA a Zorbax Eclipse XDB-C18 column (3 × 100 mm, 3.5 μm; Agilent, Waldbronn, Germany) equipped with a SecurityGuard™ column (C18, 4.0 × 3.0 mm; Phenomenex, Aschaffenburg, Germany) was used. A detailed description of the preparation and quantification process can be found in Römisch-Margl et al. [19]. The Absolute IDQ™ kit provides optimized MS instrument settings and compound parameters. System control and data acquisition was carried out with Analyst 1.5.2 software. Quantification and data evaluation was done with the MetIDQ software (version 4.5.2).

The p180 kit is validated by the manufacturer according to U.S. Food and Drug Administration guidelines [20]. However, to ensure reliability of our results, quality control (QC)-samples included in the kit were injected ten times distributed between the study samples. Additionally, in order to obtain QC samples closely related to study samples (similar matrix and concentration), we also used pooled study plasma. Six replicates of this study-specific QC sample were extracted per plate and evenly distributed amongst the study samples. AA with a coefficient of variation (CV) of >20% in the QC samples (pooled study plasma) were excluded from further analysis (glutamate and glycine), CVs for the remaining AA ranged between 6.2% and 17.3% (see Table S1). Plasma BCAA refers to the single concentrations of isoleucine, leucine, and valine plasma concentrations and not the sum of BCAA. In subsequent tables and figures, AA are presented according to three-letter codes. 

### 2.3. Dietary Assessment

Trained study personnel assessed the food intake of each individual (in g/day) on two non-consecutive days at least 4 weeks apart in a personal and a telephone interview using 24 h dietary recalls with the software EPIC-Soft [21,22]. Participants used standard units (such as slice of bread, soup bowl), household measurements (such as tablespoon) and a picture booklet providing photographs of portion sizes for various foods to indicate the consumed amount per meal. Additionally participants were requested to fill in a food frequency questionnaire (FFQ). All reported foods were then summarized into 35 food groups for further analysis (Table S2). Total energy intake (kcal/day) and intake of nutrients were estimated based on data of the German food composition database “Bundeslebensmittelschlüssel” (BLS, version 3.02) [23]. The BLS database includes data for total protein, animal-based and plant-based protein; thus, daily intake of each protein source was the sum from all food sources as well as from recipes of mixed dishes. Individual habitual intake of food groups and nutrients was estimated using the NCI method [24,25]. Within this method, habitual intake was estimated based on data of two 24 h recalls and FFQ information considering covariates age, sex, body mass index (BMI), and weekend information (Monday–Thursday = weekday, Friday–Sunday = weekend).

### 2.4. Statistical Analysis

Food groups and BCAA were not normally distributed and were therefore univariate Box—Cox transformed to approach normality [26] and z-standardized to ensure comparability prior to dietary pattern analysis. We used an optimization step to find the best transformation parameter (exponent lambda) between 0 and 2 for appropriate transformation of each variable; respective lambda values are available in Table S3. Reduced Rank Regression (RRR) was used to extract dietary patterns. RRR identifies linear functions of predictors (food groups) that explain as much response (BCAA) variation as possible [27], where the number of derived linear functions depends on the number of response variables (3 BCAA). The extracted RRR factor scores represent dietary patterns. We extracted current dietary patterns based on data of the first 24 h recall covering the past 24 h of the participants’ diet. Habitual dietary patterns were based on the calculated usual intakes representing an average of a period of more than 4 weeks of the participants’ diet. For the adjusted dietary patterns, we calculated univariate linear regression models with the RRR factor scores as dependent and age, sex, and total energy intake as independent variables. The residuals of these models represent age, sex, and energy intake adjusted dietary patterns, which were further investigated in subsequent analyses. We used partial Spearman rank correlations adjusted for age, sex, and total energy intake to investigate associations between the identified dietary pattern and the plasma AA profile. For descriptive statistics, we built quartiles of the adjusted RRR factor scores. For macronutrients we calculated their percentage of total energy intake (E%). All statistical analyses were performed using software SAS Version 9.4 (SAS Institute, Cary, NC, USA) with *p*-values < 0.05 considered as statistically significant. 

## 3. Results

### 3.1. Study Population

We excluded 11 individuals due to acute medication or illness requiring treatment. Individuals with missing information for plasma BCAA (*n* = 1) or only one available 24 h recall (*n* = 2) were also excluded from the analysis. The analytical study population includes 298 individuals, 171 men (57.4%) and 127 women (42.6%) with a mean age of 44.5 and 51.6 years, respectively. Clinical parameters were within the reference ranges. General characteristics of the study population separated by sex are shown in Table 1. 

### 3.2. Dietary Pattern Analysis

With the RRR method we derived by default a total of 6 dietary patterns (3 current and 3 habitual patterns, Table S4) that were positively correlated with the plasma BCAA concentrations. The first derived current dietary pattern covering the diet of the past 24 h, showed 19.2% of explained variance for BCAA plasma concentrations, whereas the first habitual dietary pattern, covering a period of more than 4 weeks, explained 32.5%. The remaining 4 patterns from the RRR analysis explained only a minor variation (<3.6%). Thus, we focused on the first pattern from the RRR of the habitual dietary data for all further analyses because it explains the largest amount of variation among the BCAA. 

This habitual dietary pattern correlated positively with food groups including meat, sausages, sauces, eggs, and ice cream but inversely with nuts, cereals, mushrooms, and pulses (Table 2). The first current dietary pattern was comparable with the first habitual dietary pattern showing consistent positive correlations with meat, sausages, and sauces, and inverse correlations with nuts and seeds. An overview of the average consumption of each investigated food group for the adjusted first habitual dietary pattern is shown in Table S5. 

Dividing our study group in four subgroups by the use of quartiles of the adjusted first habitual dietary pattern score, we observed slight differences with regard to the macronutrient composition. Between the lowest and highest quartile, we observed a mean difference of −2.7 E% for carbohydrates, 1.4 E% for fat and 0.8 E% for protein in the diet of the study participants (Table 3). 

At the nutrient level, we observed a statistically significant decrease of mono- and polysaccharides, of PUFAs, and of dietary fiber intakes, whereas the intake of SFAs and MUFAs was higher with an increasing adjusted habitual dietary pattern score. Over all quartiles, study participants consumed on average a higher amount of animal-based protein compared to plant-based protein. However, the percentage of animal-based protein on total protein intake increased significantly, whereas the percentage of plant-based protein intake decreased significantly with an increasing habitual dietary pattern score (Table 3).

### 3.3. Associations of Plasma Amino Acid Concentrations with Dietary Pattern Score

Of the 17 investigated proteinogenic AA, we observed eight AA to be significantly correlated with the age, sex, and energy intake adjusted habitual dietary pattern score. These included alanine, lysine, phenylalanine, tryptophan, tyrosine, and the BCAA leucine, isoleucine, and valine. The strongest correlation besides the BCAA was observed for lysine followed by the three aromatic amino acids (AAA) phenylalanine, tyrosine, and tryptophan, and alanine (Table 4). The sum of the AAA correlated as well significantly with the habitual dietary pattern score.

**Figure 1:**
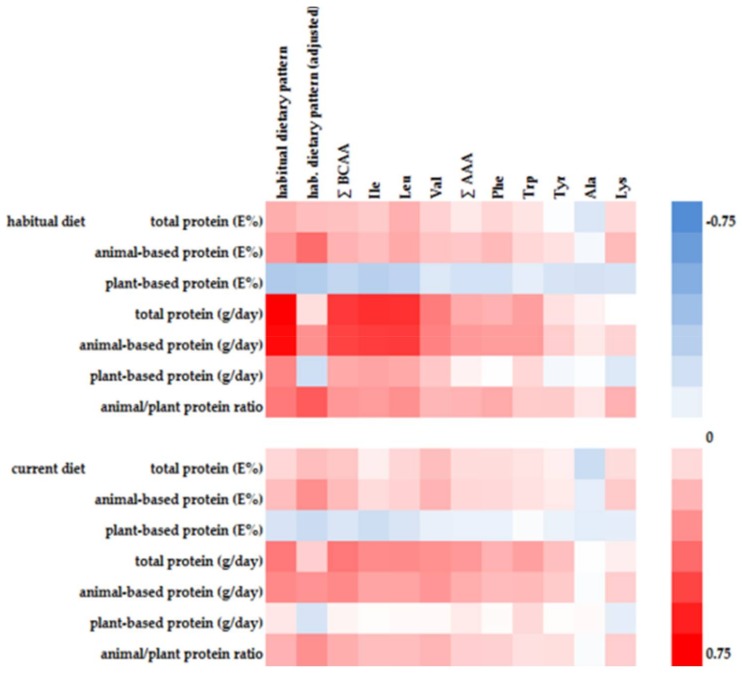
Heat map of Spearman correlations between variables of dietary protein intake and RRR-derived first habitual dietary pattern (crude and age, sex, energy intake adjusted) or selected AA, respectively.

Five of the first habitual dietary pattern associated AA showed significant positive correlations with animal-based protein intake and at the same time significant inverse correlations with plant-based protein intake (Figure 1). Alanine and tyrosine were inversely associated with plant-based protein intake, whereas tryptophan was positively associated with animal-based protein intake. 

## 4. Discussion

We have used RRR to identify dietary patterns that are correlated with plasma BCAA concentrations. Furthermore, we investigated the macronutrient and overall AA composition of the dietary pattern that explained the largest variation of plasma BCAA concentrations to identify other nutrients associated with a BCAA-rich diet. To our knowledge, we are the first group to derive an explicitly BCAA-associated dietary pattern and analyzed its composition. 

### 4.1. Current vs. Habitual Diet

There is insufficient evidence whether plasma BCAA concentrations reflect short-term or long-term dietary intake and protein intake, respectively [4,28]. Some studies observed a correlation between dietary protein intake and plasma BCAA concentrations, whereas other studies did not [16,28]. In our study the identified first habitual dietary pattern explained more than 1.5 fold percentage variation of plasma BCAA concentrations than a comparable current dietary pattern (see Table S4). Furthermore, correlations between current protein intake and plasma BCAA concentrations were consistently weaker compared to habitual protein intake (Figure 1). Consequently, circulating BCAA are likely reflecting a long-term diet or a long-term BCAA intake, respectively, which is in line with the literature, where in particular plasma concentrations of essential AA were more closely related to habitual diet [29].

### 4.2. Plasma AA Concentrations and Dietary AA Composition

Compared to other healthy study populations, our study participants showed comparable plasma concentrations for valine, but higher concentrations for leucine and isoleucine [3,30]. With regard to populations with T2D or at high risk for CVD, plasma concentrations were comparable [3] or higher [25] for leucine and isoleucine and at the same time lower for valine [3,30]. 

Plasma concentrations of the AAA were associated with the adjusted first habitual dietary pattern score in our study. Other studies reported AAA (like the BCAA) to be associated with an increased risk for T2D [31] and CVD [32]. Both, AAA and BCAA, were shown to be part of a metabolic profile that discriminates between individuals with and without metabolic syndrome. However, the total AA concentrations had only little discriminant power [33]. Clearly, a dietary pattern contributing to high BCAA and AAA levels represents more than just the sum of its single components, enclosing as well interactive effects of nutrients and other food constituents. 

Besides BCAA and AAA, two other AA were correlated with the first habitual dietary pattern score. Where BCAA have a key function in the skeletal muscle to provide nitrogen needed to maintain pools of AA such as alanine [10,34], lysine is part of the dipeptide carnitine (a nutrient high in red meat). With a carnitine and protein-rich diet, intake of lysine is increased and lysine-dependent biosynthesis of carnitine is inhibited [35]. This may further explain the positive correlation with the habitual dietary pattern in our study. As a consequence, a high intake of lysine may add to the increased risk for CVD attributed to BCAA because a carnitine-rich diet has been associated with atherosclerosis and thus CVD risk [36]. 

### 4.3. Dietary Protein Source of a BCAA-Rich Diet

Zheng et al. [1] observed markedly attenuated associations between BCAA levels and the risk of T2D when adjusting for total protein intake—indicating that the total quantity of dietary protein has a significant effect. We observed a positive correlation of the identified habitual dietary pattern with both animal-based protein intake and the animal/plant protein ratio. Furthermore, the observed associations between the dietary pattern and AA levels were robust and independent from total protein intake (data not shown). Thus, not a high protein intake in general but the protein quality may play a crucial role for modulating disease risk [16,17]. This is in line with other studies showing that not the total protein intake itself but the protein composition or the AA pattern is important with regard to risk of T2D and CVD [37,38,39]. These results strengthen our hypothesis that the dietary pattern beyond the intake of BCAA rather than the effect of BCAA intake itself contribute to the increased risk for chronic diseases. 

Dietary protein in Western diets has been reported to consist of >20% BCAA [34,40]. In our study, in particular animal-based rather than plant-based protein was correlated with the BCAA-explaining dietary pattern. This is in line with the literature, reporting BCAA levels to be highly dependent on the protein source [1]. As a consequence, plasma BCAA levels may be a surrogate marker of a long-term Western diet that is high in animal-based protein. The increased habitual intake of BCAA-rich foods may further induce a higher steady-state of BCAA (e.g., deposition in muscle tissue and higher turn-over rate) and subsequent increased plasma concentrations. 

### 4.4. Additional Nutrients of a BCAA-Rich Diet

The identified habitual dietary pattern in our study is comparable to a Western diet [41,42] that is typically high in processed foods such as meat, sausages, and foods with added sugar, and at the same time low in vegetables and fruits. There is convincing evidence that a Western dietary pattern as a whole is associated with an increased risk for chronic diseases such as T2D and CVD [43,44]. We observed small but statistically significant trends regarding the intake of some nutrients with an increasing habitual dietary pattern score. This included a higher intake of fat and in particular SFAs, accompanied by a lower intake of dietary fiber. As this pattern represents a long-term dietary intake, metabolic consequences may appear when consuming such diets over a long period. Thus, the identified BCAA-explaining habitual dietary pattern comes along with an unfavorable nutrient composition.

### 4.5. Indirect Dietary Contributions 

We observed a significant trend for a decreased intake in dietary fiber and plant-based protein paralleled by a higher intake of animal-based protein with an increasing habitual dietary pattern score. Dietary protein sources modulate risk factors for T2D and CVD [45,46]. There is convincing evidence that among others dietary fiber intake and the dietary protein source modulate gut microbiome composition [47,48,49], which additionally affects the risk for T2D and CVD [50]. Furthermore, some gut bacteria are a possible source of BCAA via microbiota dependent intestinal biosynthesis [51]. Thus, the habitual diet affects the composition and the metabolic capabilities of the intestinal microbiota, which may in turn contribute to the metabolite pattern observed in plasma.

Unfortunately, we did not assess the participants’ microbiome composition. Nonetheless, it is likely, that the observed dietary differences affect the microbiota composition and thus additionally contribute to a long-term effect regarding T2D and CVD risk. This needs to be addressed in further studies.

### 4.6. Limitations and Strengths

Our study has some limitations. Due to the cross-sectional design, we were not able to investigate any causal or temporal relationship between diet and AA profiles. We included healthy and mostly normal weight individuals. Therefore, dietary and lifestyle practices of our study participants may differ from those of the general population. Generalizing our results should be limited to groups with similar habits. As mentioned afore, we did not assess the gut microbiome composition of our study participants. 

On the other hand, this study has a number of strengths. All measures were highly standardized according to standard operating procedures. Through the use of the NCI-method, we combined information of 24 h recalls, a FFQ and diet-related covariates to investigate habitual dietary intake instead of using solely FFQ data. Due to the strict inclusion criteria, we have a very health-conscious and with regard to their diet and lifestyle very homogeneous study group without any disease or medication. Therefore, we can exclude any interference of underlying diseases/medication. We observed minor differences in the macronutrient composition which enables us to investigate differences in protein quality and origin. Within this nutritionally homogenous group, we could observe small but significant differences in the dietary composition pointing at a potentially long-term increase in disease risk. This needs to be addressed in further studies with a more heterogeneous study group. 

## 5. Conclusions

A high intake of BCAA is part of an unhealthy dietary pattern. This dietary pattern includes other risk-associated factors such as higher intake of AAA as well as saturated fatty acids, and a decreased intake of dietary fiber, all of which may contribute to the increased risk for T2D or CVD originally attributed to the BCAA.

## Figures and Tables

**Table 1:** General characteristics of the KarMeN study population.

	Men (*n* = 171)		Women (*n* = 127)	
Age (years)	44.5	±17.9	51.6	±15
Body weight (kg)	79.2	±10.2	64.3	±8.2
BMI (kg/m^2^)	24.4	±2.7	23.1	±2.9
Body fat (%)	23.0	±6.4	33.4	±6.6
Blood pressure (mmHg)				
Systolic	128.3	±14.2	120.3	±17.7
Diastolic	84.8	±10.6	83.4	±12.0
Cholesterol (mg/dL)				
HDL	62	±14	77	±16
LDL	123	±41	129	±35

Values are presented as arithmetic mean ± SD. Abbreviations: BMI, body mass index; HDL, high-density lipoproteins; LDL, low-density lipoproteins; SD, standard deviation.

**Table 2:** Partial Spearman correlation coefficients * of the RRR-derived first habitual dietary pattern score with food groups adjusted for age, sex, and total energy intake.

Food Group (g/day)	Spearmans’ rho	*p*
Sauces	0.59	<0.0001
Sausages and meat products smoked	0.58	<0.0001
Meat and meat products unsmoked	0.56	<0.0001
Ice cream	0.29	<0.0001
Eggs	0.25	<0.0001
Cereals and cereal products	−0.22	0.0001
Nuts and seeds	−0.25	<0.0001
Mushrooms	−0.26	<0.0001
Pulses	−0.29	<0.0001

* Only correlations >|0.2| are shown.

**Table 3:** AA concentrations (µmol/L) and dietary nutrient composition by quartiles of adjusted habitual dietary pattern score.

Variable	Q1 (*n* = 74)		Q2 (*n* = 75)		Q3 (*n* = 75)		Q4 (*n* = 74)		
	Min	Max	Min	Max	Min	Max	Min	Max	*p*
Adjusted first habitual dietary pattern score	−2.5	−0.43	−0.42	0.06	0.07	0.50	0.51	1.93	<0.0001
	**Mean**	**±SD**	**Mean**	**SD**	**Mean**	**SD**	**Mean**	**SD**	
Val	196.7	±51.7	198.4	±43.9	204.0	±43.4	210.2	±44.7	<0.0001
Leu	140.6	±28.5	141.3	±28.0	153.3	±32.5	161.3	±27.8	0.03
Ile	73.2	±15.3	73.8	±16.0	78.8	±18.1	83.8	±15.3	<0.0001
∑ BCAA	410.5	±86.2	413.5	±76.5	436.2	±81.3	455.3	±69.8	0.0002
Phe	55.9	±8.2	56.7	±6.7	59.0	±8.1	60.1	±8.9	0.0001
Trp	63.4	±12.8	62.8	±9.9	68.1	±13.4	67.7	±12.4	0.0010
Tyr	66.2	±13.5	67.9	±11.0	72.4	±16.1	70.3	±11.1	0.0003
∑ AAA	185.5	±28.3	187.4	±20.6	199.5	±30.3	198.2	±25.7	<0.0001
Energy intake (kcal/day)	2432	±460	2350	±464	2334	±457	2474	±497	0.90
Macronutrient intake (E%)									
CHO	45.4	±4.4	45.4	±4.5	43.9	±4.1	42.7	±4.7	<0.0001
Monosaccharides	8.1	±2.4	8.2	±2.5	7.6	±2.2	7.3	±1.9	0.02
Disaccharides	11.7	±2.2	12.4	±2.4	11.9	±2.3	11.6	±2.3	0.75
Polysaccharides	23.1	±3.1	22.5	±3.7	22.1	±3.5	21.7	±4.1	0.003
Fat	36.7	±4.1	36.7	±3.9	37.4	±3.7	38.1	±3.8	0.001
SFA	15.7	±2.5	15.9	±2.2	16.6	±2.3	16.5	±2.0	0.0001
MUFA	12.2	±1.5	12.2	±1.7	12.5	±1.4	13.1	±1.8	<0.0001
PUFA	5.9	±1.1	5.7	±1.0	5.6	±0.6	5.7	±0.8	0.047
Protein	14.3	±1.3	14.4	±1.2	14.6	±1.2	15.1	±1.5	0.0015
Animal-based protein	6.5	±1.6	7.2	±1.3	7.6	±1.4	8.4	±1.5	<0.0001
Plant-based protein	5.2	±1.0	4.9	±0.8	4.7	±0.7	4.5	±0.7	<0.0001
Animal/plant protein ratio	1.3	±0.5	1.5	±0.5	1.7	±0.5	1.9	±0.4	<0.0001
Alcohol	2.41	±1.92	2.09	±2.07	3.43	±2.96	2.95	±2.71	0.05
Dietary fiber (g/MJ)	2.8	±0.6	2.7	±0.5	2.6	±0.5	2.4	±0.5	<0.0001

*p*-Values based on Spearman correlation analysis of each variable with the adjusted first dietary pattern score. Abbreviations: AAA, aromatic amino acids; BCAA, branched-chain amino acids; CHO, carbohydrates; MUFA, monounsaturated fatty acids; SFA, saturated fatty acids; PUFA, polyunsaturated fatty acids.

**Table 4:** Spearman correlation coefficients of the RRR-derived first habitual dietary pattern with plasma AA concentrations.

	Spearmans’ rho	*p*
Ala	0.15	0.01
Arg	0.01	0.89
Asn	−0.10	0.09
Asp	0.01	0.82
Gln	−0.02	0.70
His	0.01	0.82
Lys	0.23	<0.0001
Met	0.08	0.18
Orn	0.04	0.47
Phe	0.22	0.0001
Pro	0.08	0.18
Ser	−0.03	0.67
Thr	0.04	0.51
Trp	0.17	0.003
Tyr	0.20	0.0005
Ile	0.31	<0.0001
Leu	0.33	<0.0001
Val	0.15	0.01
∑ BCAA	0.27	<0.0001
∑ AAA	0.23	<0.0001

Abbreviations: AAA, aromatic amino acids; BCAA, branched-chain amino acids.

## Supplementary Materials

The following are available online at http://www.mdpi.com/2072-6643/10/5/623/s1, Table S1: List of assessed amino acids with corresponding coefficient of variation of their respective quality control samples, Table S2: Description of food groups, Table S3: Optimized lambda-values for Box—Cox transformation of BCAA and habitual diet food groups, Table S4: RRR-derived current and habitual dietary patterns with respective factor loadings of food groups and their explained variance for plasma BCAA concentrations, Table S5: Average consumption of food groups with corresponding 95% confidence intervals by quartiles of age, sex and energy intake adjusted dietary pattern score.
