# Supplementary material for: Dietary Pattern and Plasma BCAA-Variations in Healthy Men and Women—Results from the KarMeN Study
Source: Nutrients. 2018 May 15;10(5):623. doi: 10.3390/nu10050623 (PMC5985475; doi:10.3390/nu10050623)
Supplement: Supplementary file 1 [file nutrients-10-00623-s001.pdf]

nutrients

MDPI

Supplementary Materials

Table S1. List of assessed amino acids with corresponding coefficient of variation of their respective quality control samples.

Abbreviation	LongName	CV
Ala	Alanine	6.2
Arg	Arginine	13.5
Asn	Asparagine	10.7
Asp	Aspartic acid	14.6
Cit	Citrulline	20.2
Gln	Glutamine	15.4
Glu	Glutamic acid	24.1
Gly	Glycine	21.4
His	Histidine	14.1
Ile	Isoleucine	7.8
Leu	Leucine	8.3
Lys	Lysine	9.7
Met	Methionine	11.0
Orn	Ornithine	11.6
Phe	Phenylalanine	6.7
Pro	Proline	8.8
Ser	Serine	17.3
Thr	Threonine	15.6
Trp	Tryptophan	8.8
Tyr	Tyrosine	6.3
Val	Valine	13.8

Table S2. Description of food groups.

Food group	Foods included
Wholemeal bread/rolls	
White, brown or multigrain bread/rolls	
Cereals and cereal products	Cereals, flours, rice and processed products like breakfast cereals, pasta, popcorn
Pastries	Cake, pies, savory pastries like filled puff pastries, cheese straw, snacks, peanut flips, cracker, tortilla chips
Vegetables and vegetable products	
Mushrooms	
Pulses	Including lentils, chickpeas, green peas, white/broad beans, kidney/soy beans; exception: green beans; incl. canned pulses and as sauce)
Potatoes and potato products	Fresh potatoes, heated and processed products like French fries, potato pancakes, potato chips
Fruit and fruit products (without juice)	Including unsweetened frozen fruit, fruit sauces and processed products like sweetened or heated fruit, canned fruit, dried fruit
Nuts and seeds	Hazelnuts, peanuts, almonds etc. or processed products like peanut butter, salted or roasted nuts
Animal fats	Including butter, lard, fish oil
Vegetable fats and oils	Including margarine and other spreadable fats (exception: in salad dressings)
Cheese and curd cheese	Including soft cheese, cream cheese, acid curd cheese
Milk and dairy products	Including cocoa drinks, milkshakes, yoghurt, (sour) cream, buttermilk, kefir, whey, milk powder
Eggs	Including scrambled, fried and boiled eggs, omelets, soufflé
Meat and meat products unsmoked	Including roast, goulash, schnitzel, beef olive, minced meat, meat balls, ground meat sauce
Sausages and meat products smoked	Including salami, liver sausage, ham, bacon, cured pork, meat loaf, bratwurst
Fish and fish products	Salt- and freshwater fish, shrimps, mussels, snails, processed products like caviar, tinned fish
Soups	Soups without stews
Sauces	Including warm and cold sauces (e.g. ketchup, salad dressing), mustard, vinegar etc.; exceptions: fruit-, vegetable-, ground meat sauce

Sweets	Including sweets with chocolate, confectionaries, candies, fruit gums, cereal bars
Ice cream	
Sweet spreads	Jam, jellies, honey and chocolate spreads
Desserts	Creams and desserts including pudding, semolina, tiramisu
Water	
Coffee and tea (black/green)	
Herbal/fruit tea	
Fruit juices/nectars	including spritzer
Soft drinks	e.g. lemonades, bitter lemon (incl. low-calorie drinks)
Other non-alcoholic beverages	e.g. malt coffee, malt beer, non-alcoholic beer, vegetable juices
Beer	including mixed beer drinks
Wine and sparkling wine	
Spirits	schnapps, liqueurs
Other alcoholic beverages	e.g. alcopops, cocktails
Miscellaneous	Milk substitutes (e.g. soy products (milk, yoghurt, cheese), coconut-, rice-, oat-, almond milk, cereal milk with soy), meat substitutes (e.g. tofu, tempeh, soy protein, vegetarian sausages), cereal substitutes (e.g. soy flour/flakes, lupines), sweeteners, sugar substitutes, beverage powders/ -granules (e.g. cocoa powder, lemonade powder), herbs, spices, vegetarian spreads, protein powder, yeast, miso

Table S3. Optimized lambda-values for BoxCox-transformation of BCAA and habitual diet food groups.

BoxCox-transformed variable	λ
isoleucine	0.13
valine	0.12
leucine	0.29
Wholemeal bread/rolls	0.41
White, brown or multigrain bread/rolls	0.72
Cereals and cereal products	0.09
Pastries	0.69
Pulses	0
Vegetables and vegetable products	0.63
Mushrooms	0
Potatoes and potato products	0
Fruit and fruit products (without juice)	0.87
Nuts and seeds	0
Animal fats	0.44
Vegetable fats and oils	0.08
Milk and dairy products	0.77
Cheese and curd cheese	0.79
Eggs	0.43
Meat and meat products unsmoked	0.67
Sausages and meat products smoked	0.54
Fish and fish products	0.89
Soups	0.37
Sauces	0.97
Sweets	0.55
Ice cream	0.37
Sweet spreads	0.39
Desserts	0.6
Water	0.58
Coffee and tea (black/green)	0.97
Herbal/fruit tea	0.21
Fruit juices/nectars	0.39
Soft drinks	0.21
Other non-alcoholic beverages	0.32
Beer	0.32
Wine and sparkling wine	0.36
Spirits and other alcoholic beverages	0.67
Miscellaneous	0

BoxCox-transformation: for $\lambda>0$, $x= (y^{\lambda} - 1) / \lambda$; for $\lambda=0$, $x=\log(y)$.

Table S4. RRR-derived current and habitual dietary patterns with respective factor loadings of food groups and their explained variance for plasma BCAA concentrations.

	current diet			habitual diet		
	**pattern1**	**pattern2**	**pattern3**	**pattern1**	**pattern2**	**pattern3**
explained variance of BCAA (%)	19.2	3.6	0.6	32.5	3.3	0.4
**food group**	**loading**	**loading**	**loading**	**loading**	**loading**	**loading**
Sauces	**0.50**	0.04	0.12	**0.45**	-0.14	-0.12
Meat and meat products unsmoked	**0.37**	0.16	-0.13	**0.37**	0.02	0.08
Sausages and meat products smoked	**0.39**	-0.11	-0.15	**0.34**	-0.15	0.12
Beer	**0.22**	-0.01	0.02	**0.24**	-0.14	-0.13
Soft drinks	**0.23**	-0.19	0.17	**0.21**	**-0.37**	-0.02
Desserts	-0.06	-0.02	-0.02	**0.21**	0.02	-0.05
White, brown or multigrain bread/rolls	0.09	-0.09	-0.09	0.20	-0.20	0.06
Cereals and cereal products	0.09	-0.14	0.16	0.20	**-0.21**	0.19
Eggs	0.16	0.13	**-0.25**	0.19	**0.24**	0.04
Spirits & other alcoholic beverages	0.05	**-0.21**	-0.03	0.16	-0.04	-0.06
Ice cream	0.08	**-0.34**	-0.14	0.15	**-0.24**	0.11
Vegetable fats and oils	0.18	-0.02	-0.13	0.14	-0.12	0.15
Sweets	0.05	-0.06	**-0.34**	0.13	0.04	0.19
Mushrooms	0.07	**0.20**	0.05	0.00	0.12	0.07
Fish and fish products	-0.01	**0.35**	-0.11	-0.11	**0.32**	-0.03
Coffee and tea (black/green)	-0.18	0.17	-0.04	-0.11	0.19	-0.18
Fruit juices/nectars	0.10	-0.01	**-0.20**	0.11	-0.16	0.13
Milk and dairy products	0.12	**0.38**	**0.34**	0.11	**0.25**	**-0.56**
Water	0.14	-0.14	0.19	0.10	-0.06	-0.06
Soups	-0.07	**-0.24**	0.05	-0.09	**-0.22**	-0.07
Nuts and seeds	**-0.22**	**0.29**	-0.04	-0.08	**0.21**	**-0.25**

Miscellaneous	-0.16	0.03	**-0.31**	-0.06	0.03	**0.26**
Other non-alcoholic beverages	0.06	0.14	**0.20**	0.05	0.01	-0.14
Cheese and curd cheese	0.00	-0.06	-0.07	0.04	-0.12	-0.06
Sweet spreads	-0.05	-0.06	0.02	0.04	-0.11	-0.19
Animal fats	0.02	**0.23**	**-0.28**	0.03	0.04	0.18
Potatoes and potato products	0.04	-0.06	**-0.33**	-0.03	-0.08	0.01
Wholemeal bread/rolls	-0.12	0.09	0.12	0.02	0.05	**-0.22**
Vegetables and vegetable products	0.05	0.01	-0.01	-0.02	-0.19	-0.04
Wine and sparkling wine	0.09	-0.17	-0.10	0.02	**-0.27**	-0.11
Pastries	0.03	-0.10	0.09	0.01	-0.07	0.05
Pulses	0.08	-0.07	-0.14	**-0.25**	-0.18	0.04
Herbal/fruit tea	-0.18	0.17	**-0.21**	-0.14	0.13	0.02
Fruit and fruit products (without juice)	-0.19	**0.23**	0.18	-0.14	**0.23**	**-0.40**

* Sorted according to strength of factor loadings for the first habitual dietary pattern; factor loadings >|0.2| are marked **bold**.

Table S5. Average consumption of food groups with corresponding 95% confidence intervals by quartiles of age, sex, and energy intake adjusted habitual dietary pattern score.

	Q1 (n = 74)			Q2 (n = 75)			Q3 (n = 75)			Q4 (n = 74)			
**Food Group (g/day)**	**Mean**	**LCL**	**UCL**	**Mean**	**LCL**	**UCL**	**Mean**	**LCL**	**UCL**	**Mean**	**LCL**	**UCL**	p_{trend}
Wholemeal bread/rolls	50.1	41.4	58.8	51.6	41.5	61.7	48.5	40.4	56.5	44.2	36.3	52.1	0.56
White, brown or multigrain bread/rolls	78.2	67.6	88.9	81.7	71.3	92.1	88.1	78.2	98.1	94.6	84.0	105.2	0.02
Cereals and cereal products	124	112	136	109	95	123	99	87	112	114	100	128	0.02
Pastries	64.3	58.0	70.6	68.2	60.7	75.6	59.7	53.1	66.3	63.0	56.2	69.7	0.51
Pulses	7.1	5.4	8.8	6.9	4.6	9.3	4.1	3.1	5.1	5.2	1.9	8.4	<.0001
Vegetables and vegetable products	201	184	219	194	181	207	189	176	201	186	172	201	0.04
Mushrooms	4.7	4.3	5.1	4.5	4.1	4.8	4.3	4.0	4.6	3.9	3.7	4.1	0.0001
Potatoes and potato products	57.2	50.0	64.4	52.0	45.7	58.4	59.0	52.3	65.7	52.9	46.1	59.8	0.07
Fruit and fruit products (without juice)	230	203	258	203	176	230	203	181	226	179	154	205	0.0002
Nuts and seeds	11.4	8.5	14.3	6.7	4.3	9.1	5.3	3.8	6.8	5.4	3.4	7.4	0.0001
Animal fats	9.9	7.5	12.4	8.4	6.4	10.4	9.6	8.0	11.2	7.1	5.7	8.5	0.78
Vegetable fats and oils	9.5	6.5	12.5	9.4	7.6	11.1	8.6	7.0	10.1	10.2	8.7	11.7	0.06
Milk and dairy products	183	159	207	194	169	219	184	160	208	189	168	211	0.58
Cheese and curd cheese	63.9	55.7	72.1	63.9	57.9	70.0	62.8	57.6	68.0	67.6	60.3	74.9	0.34
Eggs	14.7	9.9	19.6	13.3	11.3	15.4	14.3	12.2	16.5	19.6	15.7	23.5	<.0001
Meat and meat products unsmoked	32.0	26.2	37.8	42.6	37.1	48.2	50.9	43.4	58.3	67.7	60.6	74.8	<.0001
Sausages and meat products smoked	20.3	14.8	25.7	34.4	27.4	41.4	45.4	38.6	52.3	63.4	53.9	72.8	<.0001
Fish and fish products	17.6	15.8	19.5	17.3	15.6	19.0	15.9	14.5	17.4	15.0	13.5	16.6	0.28
Soups	60.1	47.1	73.0	41.5	32.9	50.2	57.2	45.4	69.0	48.3	37.4	59.2	0.44
Sauces	31.0	27.8	34.1	33.0	30.2	35.9	36.8	34.3	39.2	44.8	41.7	48.0	<.0001
Sweets	14.4	12.5	16.3	14.6	12.4	16.9	13.1	11.2	15.1	13.7	11.5	15.8	0.33
Ice cream	8.2	6.2	10.2	9.4	7.1	11.6	11.1	7.6	14.6	14.0	10.8	17.2	0.0002
Sweet spreads	13.6	10.6	16.7	12.0	9.3	14.7	11.2	9.0	13.3	13.5	10.2	16.7	0.86

Desserts	9.5	7.7	11.3	9.4	7.6	11.3	8.6	7.1	10.2	10.8	9.0	12.6	0.38
Water	1467	1287	1648	1331	1162	1499	1427	1268	1585	1578	1413	1742	0.86
Coffee and tea (black/green)	536	449	623	510	438	583	533	452	614	462	384	540	0.60
Herbal/fruit tea	233	150	315	225	139	311	208	123	292	136	79	192	0.14
Fruit juices/nectars	155	122	189	125	96	153	133	101	164	140	112	167	0.56
Soft drinks	40.4	22.3	58.5	100.8	42.5	159.0	48.5	17.9	79.1	94.6	50.5	138.7	0.23
Other non-alcoholic beverages	24.0	16.1	31.9	36.0	26.0	46.0	26.0	16.2	35.9	21.9	13.3	30.5	0.52
Beer	81.8	55.3	108.4	80.9	44.0	117.8	118.8	78.7	158.9	109.3	75.0	143.6	0.15
Wine and sparkling wine	47.0	35.1	59.0	41.7	28.8	54.5	51.2	35.0	67.4	46.4	33.3	59.4	0.42
Spirits and other alcoholic beverages	1.2	0.9	1.4	1.0	0.8	1.2	1.2	0.9	1.5	1.4	1.1	1.6	0.11
Miscellaneous	43.2	21.8	64.5	16.3	8.5	24.0	19.8	9.2	30.4	8.0	4.7	11.3	0.04

Abbreviations: LCL, lower 95% confidence limit; UCL, upper 95% confidence limit.
